# Immunity to Infections after Haploidentical Hematopoietic Stem Cell Transplantation

**DOI:** 10.4084/MJHID.2016.057

**Published:** 2016-10-25

**Authors:** Franco Aversa, Lucia Prezioso, Ilenia Manfra, Federica Galaverna, Angelica Spolzino, Alessandro Monti

**Affiliations:** Hematology and BMT Unit, Department of Clinical and Experimental Medicine, University of Parma, Parma, Italy

## Abstract

The advantage of using a Human Leukocyte Antigen (HLA)-mismatched related donor is that almost every patient who does not have an HLA-identical donor or who urgently needs hematopoietic stem cell transplantation (HSCT) has at least one family member with whom shares one haplotype (haploidentical) and who is promptly available as a donor. The major challenge of haplo-HSCT is intense bi-directional alloreactivity leading to high incidences of graft rejection and graft-versus-host disease (GVHD). Advances in graft processing and pharmacologic prophylaxis of GVHD have reduced these risks and have made haplo-HSCT a viable alternative for patients lacking a matched donor. Indeed, the haplo-HSCT has spread to centers worldwide even though some centers have preferred an approach based on T cell depletion of G-CSF-mobilized peripheral blood progenitor cells (PBPCs), others have focused on new strategies for GvHD prevention, such as G-CSF priming of bone marrow and robust post-transplant immune suppression or post-transplant cyclophosphamide (PTCY). Today, the graft can be a megadose of T-cell depleted PBPCs or a standard dose of unmanipulated bone marrow and/or PBPCs. Although haplo-HSCT modalities are based mainly on high intensity conditioning regimens, recently introduced reduced intensity regimens (RIC) showed promise in decreasing early transplant-related mortality (TRM), and extending the opportunity of HSCT to an elderly population with more comorbidities. Infections are still mostly responsible for toxicity and non-relapse mortality due to prolonged immunosuppression related, or not, to GVHD. Future challenges lie in determining the safest preparative conditioning regimen, minimizing GvHD and promoting rapid and more robust immune reconstitution.

## Introduction

Haploidentical hematopoietic stem cell transplantation (haplo-HSCT) is a valuable treatment option for patients with various hematological disorders who lack a suitable HLA matched donor or for whom an HSCT is urgently required.^1^–^3^ Actually, the road to full maturity of haplo-HSCT was beset by clinical problems. Until the early 1990s, haplo-HSCT was associated with a high incidence of graft rejection in T-cell–depleted transplants and severe graft-vs-host disease (GVHD) in unmanipulated transplants because of the high frequency of T cells that recognized major class I or II HLA disparities between donor and recipient.^3^–^5^ To overcome these problems, two approaches were developed: a megadose of T-cell–depleted hematopoietic progenitor cells without any post-transplant immunosuppression^4^,^6^,^8^,^9^ and unmanipulated grafts with innovative pharmacological immunosuppression for GVHD prophylaxis.^3^,^5^,^10^–^12^

Whereas the use of reduced intensity conditioning (RIC), infusion of mega doses of CD34+ cells, and graft manipulations such as selective T cell depletion were helpful to achieve engraftment with lower rates of GvHD and toxicity, delayed immune reconstitution and infectious complications remain outstanding issues for haplo-HSCT and are important causes of morbidity and mortality.^3^,^7^,^10^,^12^,^13^ In the early post-transplant period, neutropenia is the principal risk factor for infections while, once engrafted, the capacity to mount an adaptive immune response to pathogens is a key factor for protecting from severe and recurrent infectious complications.

This review describes the most common infectious complication of the haploidentical HSCT and the mechanisms that may have a role in the incidence and severity of these complications.

## Post-Transplant Immunological Reconstitution and Infections

Reconstitution of the T-cell pool after HSCT is achieved both through peripheral expansion of naïve and memory T-cells,^14^ and de novo differentiation of hematopoietic stem cells in the thymus.^15^ T-cells originating from peripheral expansion would most likely have a more limited TCR repertoire. They could also, at least in theory, be more allo-reactive, not having gone through the process of negative selection in the recipient. In adults, due to the decay in thymus function, post-grafting immune recovery depends, for months, on the expansion of the mature T cells infused with the graft. Naıve T cells are produced months after transplantation because conditioning induced tissue damage prevents T cell homing to peripheral lymphoid tissues, where T cell memory is generated and maintained.^17^ Furthermore, the post-HSCT adaptive immune response is influenced by the strategy used to prevent GvHD.^3^,^6^,^13^ In unmanipulated haplo-HSCT, peripheral T-cell expansion is antagonized by the immune suppressive therapy for GVHD prophylaxis. In T cell depleted haplo-HSCT the T-cell repertoire is very narrow since the number of T lymphocytes in the graft has to be particularly low to prevent GvHD, and anti-thymocyte globulin (ATG) in the conditioning exerts an additional in vivo T-cell depletion.^13^,^18^ Even in the absence of pharmacologic agents, GVHD itself is known to have deleterious effects on immune function and can cause profound lymphoid hypoplasia, B cell defects and damage to thymic stroma, resulting in impaired T cell development.^19^ Thus, the immune recovery is slow, and patients tend to remain susceptible to opportunistic infections for several months after HSCT. In a recent review, Fabricius and Ramanathan^3^ reported data obtained from retrospective comparative studies by Raiola et al.,^20^ nCiurea et al.,^21^ and Gragert et al.^1^ nand a prospective study by Wang et al..^22^ The incidence of infection complications after haploHSCT compares favorably with that reported after any other HSCT from alternative donors (Table 1). The rate of fatal infections were 11% in haplo, 14% in MUD, 17% in UCB and 4% in MSD.

### Bacterial infections

The incidence of bacterial infections varies according to transplant phase, preparative conditioning regimens, underlying disease, disease status, patient’s age and comorbidities.^23^ Severity and duration of the neutropenia, the presence of central venous catheters and mucositis are still the most important risk factors for bacterial infections during the pre- and peri-engraftment periods. On the other hand, the incidence of bacterial infections in the post-engraftment phases is low. The intensity of the conditioning regimen plays a role in the risk of bacterial infections. Using a reduced intensity conditioning (RIC) regimen instead of a myeloablative (MA) protocol significantly reduces the severity of the mucosal damage as well as the length and the severity of the neutropenic phase and consequently the risk of the translocation of bacteria into systemic circulation. A recent review by Balletto and Mikulska on epidemiology and incidence of bacterial infections after haplo-HSCT confirmed that bloodstream infections (BSIs) are the most frequent documented bacterial infections followed by pneumonia and gastrointestinal infections, including typhlitis and infections due to Clostridium difficile.^23^ However, defined data about the cumulative incidence of bacterial infections in haplo-HSCT are lacking, due to the presence of various haplo-HSCT platforms that differ substantially from each other.

### Gut microbiota

Microbial communities in the gut are important in protecting the host against pathogenic microbes. Notably, gut microbiota plays important roles in the normal architecture of secondary lymphoid organs, differentiation of induced regulatory T cells and generation of IgA-secreting B cells as well as of memory alloreactive T cells.^24^,^25^ In physiological condition the gut microbiota is essential to promote mucosa integrity and downregulate pro-inflammatory cytokines^26^,^27^ while disruption of microbiota is potentially responsible for other infections.^28^ During allo-HSCT, the diversity, and stability of the intestinal microbiota are disrupted and became dominated by bacteria associated with subsequent bacteremia.^29^ Several studies have shown that gut microbial dysbiosis may have a link with complications after HSCT, including GVHD. In mouse models and patients with GVHD after BMT, Jenq et al.^30^ observed a loss of microbial diversity and Clostridiales and expansion of Lactobacillales in intestinal microbiota. Eliminating Lactobacillales from the gut flora in mice before BMT could cause GVHD. When reintroducing a predominant species of Lactobacillus, GVHD was alleviated. After HSCT, a relative shift toward Enterococci in intestinal microbial communities was also found. Specifically, the shift was prominent in patients who subsequently developed or suffered from active gastrointestinal GVHD.^31^ Interesting evidence of intestinal dysbiosis in HSCT recipients could be exemplified by increasing the incidence of Clostridium difficile infection (CDI) after allo-HSCT. A better understanding of the relationship between gut microbiota and complications after allogeneic HSCT may make gut microbiota as a therapeutic target in the future.^25^

### Invasive fungal infection

Haplo-HSCT recipients are at high risk of invasive fungal infection (IFI) involving Candida and Aspergillus species.^32^,^33^ In studies focusing on IFI, alternative transplants are described together with other allogeneic transplants, but the incidence rate of IFI may vary considerably from 7.3% in HLA-identical sibling to 27% in alternative HSCT recipients.^32^–^34^ The epidemiological findings of IFI have been longer reported in retrospective studies, and only few prospective series have been recently published.^32^ The Italian Cooperative Group [Gruppo Italiano Trapianto Midollo Osseo (GITMO)] conducted a large, multicenter, prospective epidemiological study on 1858 patients.^33^ The cumulative incidence of proven or probable IFI was 5.1, 6.7 and 8.8% at 40 days, 100 days and 12 months from transplant, respectively. Invasive aspergillosis was the most common infection (81.1%), followed by invasive candidiasis (11.0%), zygomycosis (3.7%) and fusariosis (1.8%). This study demonstrated that patient populations with different types and severity of GVHD and with different donors are at significantly different infectious risks. The cumulative incidence of IFD in patients with acute GVHD, not followed by a chronic GVHD, was 2.3% and 10% after transplant from matched related donor and alternative donors, respectively. The infectious risk became higher when acute GVHD was followed by chronic GVHD, as 10% of transplants from matched-related donor and 25% of those grafted from alternative donors were complicated by an IFD. Of interest, the cumulative incidence of IFDs was relatively low (<4%) in patients with a chronic GVHD not preceded by an acute GVHD, also named ‘de-novo’ chronic GVHD, regardless of the type of donor. As expected, the risk for IFD was significantly higher in patients with grade III–IV as compared with those with grade II acute GVHD.^33^ Actually, the most recent data on unmanipulated haplo-HSCT show that IFI does not represent a relevant posttransplant complication as compared to MUD and UCB transplants that remain at higher risk for IFI, particularly invasive aspergillosis (Table 2).^35^–^39^

### Viral infection

Reactivation of latent viruses such as cytomegalovirus (CMV), Epstein Barr virus (EBV), herpes simplex virus (HSV) and varicella zoster virus (VZV) commonly causes symptomatic disease. CMV and EBV may cause pneumonia and post-transplantation lymphoproliferative disease (PTLD), respectively.^41^,^42^ Despite the use of prophylactic and preemptive treatment, CMV-related infection continues to affect outcome of haplo-HSCT and represents one of the main causes of morbidity and mortality.^43^–^45^ The combination CMV donor-negative into CMV recipient-positive is associated with the highest risk for CMV infection and disease.^45^ In a retrospective study based on the EBMT registry database, CMV seropositive patients receiving seropositive unrelated donor grafts had reduced NRM and improved survival compared to those receiving seronegative grafts.^46^

Although surveillance and therapeutic strategies have been established for several viral infections (including CMV infection), such strategies are not yet established for adenovirus (AdV) infection. AdV infection, usually resulting from reactivation of the virus,^47^ can be divided into three categories: asymptomatic AdV infection, localized AdV disease (e.g., hemorrhagic cystitis (HC), colitis, and pneumonia), and disseminated AdV disease. The reported mortality of patients with disseminated AdV disease is remarkably high, varying from 20 % to 80 % in different studies.^47^–^49^ Due to the low incidence of AdV viremia in the absence of clinical symptoms no consensus exists on whether systematic surveillance for AdV viremia should be performed for adult patients at high risk.^50^ Taniguchi and coll^48^ in Japan observed a high incidence 85.8%) of disseminated AdV disease in adult patients undergoing unmanipulated haplo-SCT whose immunologic reconstitution was compromised by the use of ATG and steroid as GVHD prophylaxis. Disseminated AdV disease was always associated with HC. Ethnicity appears to have an influence on these results because Japanese patients have a higher incidence of latent infection of group B AdV, which can translate into a higher incidence of AdV-associated HC. Overall, AdV commonly causes severe morbidity and mortality in children while the incidence of ADV infection in adult patients is low and so the usefulness of routine monitoring of AdV DNA in asymptomatic patients remains uncertain.^51^ Other viral infections, such as the polyoma BK virus (BKPyV) contribute to the occurrence of HC.^52^ HC is associated with significant and prolonged morbidity, especially in highly immune compromised patients, increasing hospitalization duration and, consequently, the cost of transplant procedures.

Infections due to HHV-6 are generally encountered earlier than CMV. HHV-6 may lead to engraftment delays or graft failure. It may also cause a prominent disease with a facial rash and confused with encephalitis or acute GvHD.^53^,^54^

### Parasitic Infections

The incidence of toxoplasmosis in allo-SCT recipients is high in areas of endemicity, ranging from 6% in Europe and 3% in Brazil compared to 50.5% in USA or Japan.^55^ Patients with toxoplasmosis can present with fever, lymphadenopathy, hepatosplenomegaly, meningitis, brain abscess, chorioretinitis, pneumonitis, myocarditis, hepatitis, pancytopenia or disseminated disease. Symptoms often present within 3 months post-transplant, but later presentations may occur, particularly after discontinuation of chemoprophylaxis, or even interruption of the anti-pneumocystis prophylaxis with cotrimoxazole, which may exert a protective role.^56^,^57^ Diagnosis can be difficult in the absence of typical ring enhancing brain lesions on computerised tomography or magnetic resonance imaging scans in some cases. The highest risk patients are seropositive allo-SCT recipients who have received cord blood or unrelated donor transplant, T cell depleted transplants.

### T Cell Depleted HaploHSCT

In general, TCD techniques can be classified as *in vitro* if the stem cell manipulation is performed ex vivo exclusively, normally by column adsorption. In contrast, *in vivo* techniques are based on a partial or complete depletion of donor lymphocytes in the patient after transplanting the stem cell product using ATG or alemtuzumab. In the early ‘1990s, Aversa et al. exploited the principle of a megadose T cell depleted HaploHSCT in patients with acute leukemia and showed that an extensive ex vivo T-cell depletion followed by the infusion of a mega-dose of immune-selected CD34+ cells prevents both graft rejection and GvHD even in the absence of post-transplant immunosuppression.^8^,^9^ However, due to the delayed immunological reconstitution, infectious complications remained an issue, in particular with a focus on viral infections in the early and intermediate posttransplantation phase. In their analysis of 103 patients with high-risk acute leukemia, 27 of the 38 non-relapsing deaths were caused by infections, whether viral (14 CMV, 1 HHV6, 1 adenovirus, 1 EBV), fungal (4 Aspergillus fumigatus, 1 Candida albicans), or bacterial (2 Pseudomonas aeruginosa, 2 Streptococcus viridans, 1 Escherichia coli).^58^ The Swiss Blood Stem Cell Transplantation group retrospectively reviewed the haplo-HSCT performed in Switzerland from 1998 to 2010 with the aim of analyzing the effect of in vitro TCD with graft engineering (CD34 selection or CD3/CD19 depletion, 74%) or in vivo TCD using alemtuzumab (26%) on immune reconstitution and infections.^59^ Despite anti-infective prophylaxis, most patients (94%) experienced post-transplant infectious complications, 11 patients had four infections. The overall incidence of bacterial infections was 56/69 (81%), most frequently due to gram-negative bacteria (26%) and staphylococcal infections (16%). Fungal infections occurred in 22/69 patients (32%) and viral infections in 45/69 (65%) patients, mostly CMV (35%), herpes (24%) and BK (23%) virus infections. Patients with in vivo TCD had a higher incidence of CMV reactivations (54% vs. 28%, p= 0.015), but this did not result in a higher NRM. The main reasons for NRM were acute respiratory distress syndrome (n=4), viral infections (n=3), pulmonary aspergillosis (n=2), Pneumocystosis (n=1), sepsis (n=2), toxoplasmosis (n=1), GVHD (n=1) and myelitis (n=1). This study confirms that alemtuzumab represents an important risk factor for CMV reactivation in patients receiving haplo-HSCT.^60^

As the Achilles heel of T cell depleted haploHSCT was linked to the paucity of T lymphocytes in the graft, over the past decade, various strategies of adoptive donor T-cell immunotherapy have been investigated to improve immune recovery and reduce non-relapse mortality (NRM) from infectious complications.

### Infusion of Pathogen-Specific T Cells

Some groups have focused on the adoptive transfer of pathogen-specic T lymphocytes against CMV, aspergillus, adenovirus and EBV. In the original study by Perruccio et al.,^61^ large numbers of donor pathogen-specific T-cell clones were generated, then screened individually for alloreactivity against recipient cells, deleted of those cross-reacting against recipient alloantigens, and infused soon after haplo-HSC. Infusion of Aspergillus-specific type-1 CD4+ clones controlled Aspergillus antigenemia and helped to clear invasive aspergillosis in 9 of 10 patients. Similarly, infusion of CMV-specific CD4+ clones largely prevented CMV reactivation and reduced CMV mortality. Since clearance of virally infected cells is mediated by specific CD8+ cytotoxic cells, the infused CD4+ cells might have conditioned APCs to stimulate the CMV-specific CD8+ T cells transferred with the graft, thus promoting their clonal expansion. In fact, unlike non-infused control patients, CMV-specific CD8+ cells were detected shortly after infusing CMV-specific CD4+ clones. Among patients receiving T-cell therapy, total CD4+ and CD8+ T-cell counts were significantly higher. The successful transfer of immunity to Aspergillus and CMV triggered neither acute nor chronic GvHD.^61^

An alternative to pathogen-specific therapy is adoptive T-cell immunotherapy, which provides large numbers of wide repertoire cells, mirroring the physiologic immune system. The key challenge is to infuse sufficient T cells without causing GVHD. Strategies include broad repertoire T cells depleted of alloreactive T lymphocytes or engineered with a suicide gene.

### Ex Vivo Photodepletion of Alloreactive Donor T Cells

Photodynamic purging appears to be an effective strategy for selectively depleting donor alloantigen-specific T cells, thus preventing GvHD and preserving the T cell anti-leukemia function. In a mixed lymphocyte reaction, alloantigen-stimulated T cells uptake 4,5-dibromorhodamine methyl ester (TH9402), a compound that is structurally similar to Rhodamine.^62^ The study by Perruccio et al.,^63^ investigated a range of parameters, and combinations thereof, with the aim of achieving optimal T cell allodepletion and preservation of pathogen-specific responses. The remarkable drop in frequency of alloreactive T cells is expected to allow safe infusion of relatively large numbers of T cells across histocompatibility barriers for adoptive transfer of donor immunity. Patients up to age 62 years with high-risk hematologic malignancies were enrolled in a phase-1 dose escalating study.^64^ All patients engrafted rapidly, and no severe acute GVHD occurred in the absence of immune suppressors. Higher doses were associated with lower TRM and improved survival. This effect was mainly attributed to a decrease in infectious complications and low relapse rates. These findings led to the initiation of an international multicenter phase II clinical trial and, at interim analysis, patients receiving 2×10^6^/kg photodepleted CD3+ T cells did not have severe GVHD and demonstrate a high overall survival (69% at 12 months after HSCT).^10^

### Infusion of T Cells Engineered to Express Suicide Genes

Polyclonal T cells were engineered to express suicide genes, eg, the herpes simplex thymidine kinase (HSV-TK) gene, to guarantee engineered cell lysis if they triggered GvHD.^65^–^68^ Ciceri et al. reported the results in a cohort of 50 high-risk leukemia patients enrolled in phase I–II, multicentre, non-randomised trial.^67^ Overall, there were 196 infectious events (median four events per patient, range 0–14), 161 of which occurred with 130 days. In immune reconstituted patients, progressive normalization of antiviral responses was associated with a decline in the number of infectious events, while patients who failed immune reconstruction continued to have frequent infectious complications. After 130 days, median peaks in blood titres of CMV antigen were 0 nuclei per 10 peripheral blood mononuclear cells (PBMC) (range 0–20) in immune reconstituted patients and 21 nuclei per 10 PBMC (range 14–58) in patients without immune reconstitution (p<0·0155); and median length of antiviral treatment was 0 days (range 0–44) in immune reconstituted patients and 47 days (range 33–105) in patients without immune reconstitution (p<0·0052). The conditional benefit of immune reconstitution obtained by TK-cell infusion was assessed by the cumulative incidence of non-relapse mortality for patients alive 100 days after transplant; non relapse mortality was 14% (infectious mortality 9%) in TK-treated immune-reconstituted patients and 60% in non-immune-reconstituted patients. A randomized phase III trial to address the role of HSV-TK donor lymphocyte addbacks for recipients of haplo-HSCT is ongoing at present.

Other researchers devised an inducible T-cell safety switch based on the fusion of human caspase 9 to a modified human FK-binding protein, allowing conditional dimerization and cell suicide following administration of the small molecule dimerizing drug AP1903.^69^ Since preliminary interesting results, the Rome group has recently launched a phase I/II study enrolling children with either malignant or nonmalignant disorders who will receive TCR-αβ/B cell depleted HaploSCT, followed by the infusion of titrated numbers of iC9 T cells on day 14±4. These iC9-modified T cells can contribute to T cell immune reconstitution after T cell depleted HaploSCT and are eliminated by the administration of AP1903, if aGVHD occurs.^70^

### Regulatory T Cells

More recently, a pioneer experience of the Perugia group has clearly demonstrated that naturally occurring Tregs harvested from healthy donors efficiently control the alloreactivity of a large number of otherwise lethal, conventional T cells.^71^–^73^ Using this strategy, there was a rapid, sustained increase in peripheral blood T-cell subpopulations. A wide T-cell repertoire developed rapidly. Naïve and memory T-cell subsets increased significantly over the first year after transplantation, demonstrating sustained immune recovery over time. B-cell reconstitution was rapid and sustained and immunoglobulin serum levels normalized within 3 months. Compared with standard haplo-HSCT, specific CD4+ and CD8+ for opportunistic pathogens such as Aspergillus fumigatus, Candida albicans, CMV, ADV, HSV, and toxoplasma emerged significantly earlier, fewer episodes of CMV reactivation occurred, and no patient developed CMV disease. Nevertheless, 8 of the 13 non-relapse deaths were due to infections: adenoviral infection (n=2), bacterial sepsis (n=1), toxoplasmosis (n=1), fungal pneumonia (n=3) or central nervous system aspergillosis (n=1).

### Selective T cell depletion

Other attempts to improve post-transplant immune recovery focused on improving graft content by shifting from CD34-positive selection to negative selection of PBPCs so as to include other immune cells.^10^,^74^ Selective T-cell removal means depletion of a given subset of the whole T-cell population. The aim is to reduce the incidence of GvHD while preserving other beneficial cell functions carried out by the residual T-cell subsets. In an innovative approach, Handgretinger’s group in Tubingen depleted the leukapheresis product of only TCR αβ+ T cells, thus retaining large numbers of effector cells such as TCRγδ+ T cells and NK cells.^75^,^76^ TcRγδ+ T cells combine conventional adaptive features with direct, rapid responses against sterile stresses and many pathogens. They participated in the anti-CMV response in the early period of post-transplant immune recovery. They are not expected to initiate GVHD because they do not recognize specifically processed peptide antigens as presented on major histocompatibility complex (MHC) molecules. First clinical results of these new T-depletion strategies are encouraging and interestingly none of the studies reported a significantly increased incidence of infections, even using MAC regimens.^75^–^78^ This could be partially explained by the high number of γδ T cells in donor’s graft. Indeed, γδ T cells are considered as a bridge between adaptive and innate immunity. γδ T cells receptors detect unconventional antigens such as phosphorylated microbial metabolites and lipids, non-classical MHC-I molecules and unprocessed proteins.^79^ They are concentrated within epithelial and mucosal surfaces to maintain the epidermal integrity of the skin and intestinal epithelium.^80^ It has been hypothesized that tissue-specific antigens are recognized by γδ T-cells resulting in immune responses protecting potential sites of pathogen entry into the body.^81^

In two cohorts of children transplanted either in Tubingen^75^,^76^ or in Roma,^77^,^78^ no post-transplant GVHD prophylaxis was given. Engraftment was very rapid in all patients. Few had acute grade I–II GVHD, and none developed chronic GVHD. Immune reconstitution was fast. Locatelli et al.^77^ prospectively assessed functional and phenotypic characteristics of γδ T lymphocytes up to 7 months after haplo-HSCT depleted of αβ+ T cells and CD19+ B cells in 27 children with either malignant (n=15) or nonmalignant disorders. Notably, in patients that experienced CMV reactivation they observed a significant expansion of Vδ1 T-cell subset; these subsets display a cytotoxic phenotype and degranulate when challenged with primary acute myeloid and lymphoid leukemia blasts. These results have been recently confirmed in 23 children with non-malignant disorders.^82^ The cumulative incidence of grade 1 to 2 acute GVHD was 13.1%. None of the 21 patients at risk developed chronic GVHD. The 2-year DFS was 91%. Two died of infectious complications (one CMV-related pneumonia and one disseminated adenovirus infection) 120 and 116 days after HSCT, respectively. Overall, 9 children experienced viral infections and/or reactivations, the cumulative incidence of CMV and adenovirus infection being 38%. Nevertheless, the cumulative incidence of TRM was 9%.

Perko et al.^83^ recently investigated immunological reconstitution of 102 pediatric patients with acute leukemia who underwent HSCT in first complete remission, focusing on the potential role of γδ T-cells. They found that γδ T cell recovering during the first year after HSCT correlated with a reduced incidence of infection. Indeed, patients with an elevated number of γδ T cell experienced only viral infection, while low/normal γδ T cell group had viral, bacterial and fungal infections; cumulative incidence of bacterial infection was 0% vs. 26.4%, respectively. Enhanced γδ T cell recovery resulted in higher EFS rate at 1 year. A possible reason to explain these results could include faster reconstitution of intestinal mucosa integrity, or prompt anti-infective function of γδ T cell, and possibly a better balance within gut microbiota.

To determine whether the post-transplant immunological reconstitution can be improved even in adult patients, this approach was recently tested in 38 adult patients, median age 35 years (range 19–73), with acute leukemia (n=28), lymphoma (n=4) or others diseases (n=6).^84^ Conditioning included ATG, Treosulfan, Fludarabine, and Thiotepa. No pharmacologic prophylaxis for GvHD was given after transplantation. Grafts contained a median of 11,6 ×10^6^/kg (range 5–19) CD34+ cells, 4 ×10^6^ CD3+ T cells/kg (range 1–35), 4,4 ×10^4^/kg (range 0.4–62) αβ+T cells/kg, 3,85 ×10^6^ γδ+ Tcells/kg (range 1–34), 4,9 ×10^4^ B cells/kg (range 1.8–32) and 23,40 ×10^8^ CD56+NK cells/kg (range 8–91). All patients but one achieved rapid, sustained full donor-type engraftment, with a median time to reach 500 neutrophils and 20,000 platelets of 12 (range 10–18) and 11 days (range 6–16), respectively. Overall, acute GvHD occurred in 6 patients. Only one patient, who had received the highest dose of αβ+ T cells (3.7×10^5^/kg), developed and died from grade III–IV acute GVHD. Skin limited acute GvHD occurred in the remaining 5 patients. Only one patient progressed to moderate chronic GvHD. Tending to confirm the working hypothesis, there was a rapid, sustained increase in peripheral blood T-cell subpopulations. The CD4 and CD8 counts reached 200/μL medianly on, respectively, days 45 (range, 19–98) and 38 (range, 13–69). Naïve and memory T-cell subsets increased significantly over the first year after transplantation. B-cell reconstitution was rapid and sustained and immunoglobulin serum levels normalized within 3 months. A single CMV reactivation occurred in 4 cases (in only one with unfavorable CMV-serology, CMV recurrence was documented 3 times), no patient has so far developed CMV disease. In two patients, CMV reactivation was associated with a significant expansion of pathogen-specific CD8+ T cells and both cleared viral load spontaneously. No patient had Epstein-Barr virus-related post-transplantation lymphoproliferative disease, and no invasive fungal disease occurred. The cumulative incidence of NRM was 20% even though 11 patients were in the upper age for transplantation (between 61 and 73 years). Overall, six of the 11 non-relapse deaths were due to infections: 1 EBV, 1 adenovirus, 1 RSV, 1 miliary tuberculosis, 2 gram-negative sepsis.

All these recent experiences confirm that current T cell-depleted HSCT strategies (either Treg/Tcon immunotherapy or αβ T cell depletion) offer the unique opportunity to harness both natural and adaptive immunity to control leukemia relapse and infections in the absence of GvHD.

#### Unmanipulated haploHSCT

Crossing the histoincompatibility barrier in HSCT is today feasible without *ex vivo* T- cell depletion. Two major approaches have been so far used: the GIAC-based strategy and the posttransplant CY-based protocol.

### The “GIAC” Strategy

This modality is based on the following four elements: (G) donor treatment with recombinant granulocyte colony-stimulating factor (rhG-CSF); (I), intensified immunologic suppression; (A), (ATG; (C), a combination of PBPCs and bone marrow cells. In the original study, Huang et al.^85^ reported the results in 171 patients who had received a myeloablative conditioning and intensive posttransplant immunosuppression that included ATG, cyclosporine, methotrexate, mycophenolate mofetil, and anti-CD25 antibody (basiliximab). All patients achieved sustained, full donor chimerism. The 2-year incidence of opportunistic infections was 40%. In their most recent update including 250 acute leukemia patients, a total of 120 occurrences of opportunistic infections were recorded in 106 patients during the duration of follow-up.^86^,^87^ The median time for an opportunistic infection to develop was 280 days (range, 5–1120) after transplantation. The infected loci included lungs (74 occurrences), skin (28 occurrences), gastrointestinal tract (24 occurrences), and central nervous system (6 occurrences). Causes of infections of the skin were varicella-zoster (16 cases) and herpes simplex virus (12 cases). Pneumonia was due to bacteria in 13 cases, Aspergillus in 18 cases, Candida albicans in 1 case, Pneumocystis carinii in 5 cases, and CMV in 16 cases. In the other 21 cases, no pathogen could be documented, and 9 of them responded to antibiotics. At 3 years after transplantation, the cumulative incidence of opportunistic infections was 49.1%. The cumulative incidence of grade III–IV aGVHD was 13.4%, the incidence of cGvHD and extensive cGVHD at 2 years was 54% and 22.6%, respectively. Even though a higher disease-free survival was achieved–partly due to the inclusion of standard and good risk patients - the concern remains that a higher incidence of GVHD is usually associated with a higher treatment-related mortality and higher cost of care for these patients.

Consistent with their previous work, the Benjing group showed that high-dose ATG was associated with delayed recoveries of CD19+ B cells, CD3+ T cells, and CD4+ T cells during the first month after haploHSCT.^88^ Furthermore, they also showed that high-dose ATG delayed the recoveries of CD4+, CD4+CD45RA+, and CD4+CD45RO+ T cells for 2 months, delayed the recovery of CD4 CD8 T cells for 6 months, and delayed the recovery of CD8+CD28+ T cells for 12 months after transplantation. The persistent delay in CD4 CD8 T cell recovery was closely related to an increased risk of EBV infection post-haploHSCT. The study showed that the schedule based on 6 mg/kg ATG was associated with a faster recovery of T cell subsets and a lower incidence of EBV infection compared to the schedule of 10 mg/kg ATG.

Using the Peking-based strategy, Di Bartolomeo et al.^36^ yielded promising results in 80 acute leukemia patients (median age of 37 years, range, 5–71). A myeloablative conditioning (MAC) regimen was used in 64 (80%) patients and a reduced intensity conditioning (RIC) in the other 16 (20%). They achieved a 91% engraftment rate, with a median of 21 days (range, 12–38) for the absolute neutrophil count and 28 days (range, 14–185) for platelets. The cumulative incidences of grade 2–4 aGVHD and cGVHD were 24% and 17%, respectively. Twenty-seven patients (34%), 13 in the standard-risk group and 14 in the high-risk group, respectively, died from transplantation related complications at a median time of 76 days (range, 6–369). Causes of death included infections in 11 patients (14%), pneumonia in 5 (6%), multiorgan failure in 5 (6%), acute GVHD in 3 (4%), liver failure in 1 (1%), veno-occlusive disease in 1 (1%), and CNS disease complications in 1 (1%). TRM was 32% at 6 months and 36% at 1 and 3 years. In the first 6 months after transplantation, 56 patients developed CMV reactivation (C.I. 70%), 38 bacterial septicemia (C.I. 47%), 25 hemorrhagic cystitis (C.I. 31%), 13 CNS complications (C.I., 16%), 10 fungal infections (C.I. 14%), and 5 veno-occlusive disease (C.I., 6%). The 3-year probability of OS for all patients was 45% (54% for the standard-risk group and 33% for high-risk group (P=06).

Arcese et al.^89^ have recently updated the results of 97 patients who received a single conditioning regimen, even though with different intensity according to age and comorbidity (TBF-MAC=68; TBF-RIC=29), before the infusion of an unmanipulated G-CSF-primed BM from a haploidentical donor. Regardless of the conditioning regimen, the GvHD prophylaxis was identical for all the patients and included five drugs: ATG, CSA, MTX, MMF and the anti-CD25 monoclonal antibody (basiliximab). Neutrophil and platelet engraftment rates were 94% and 84%, respectively. The cumulative incidence of grade II–IV acute and extensive chronic GvHD was 31% and 12%, respectively. Overall, 31 patients (32%) died of transplant-related complications at a median of 76 days (range 9–527). The infections were the leading cause of NRM accounting for 48% of all deaths. At 1 and 5 years, NRM was 31% and 34%, respectively.

### Post-transplantation Cyclophosphamide

The most commonly used new strategy for GvHD prevention is in vivo depletion of bidirectional alloreactive T lymphocytes by means of high doses of post-transplant CY (PTCY).^2^,^5^,^10^–^12^ Indeed, PTCY preferentially targets proliferating alloreactive T cells that are activated in vivo after recognition of alloantigen, thus reducing the risk of both GvHD and graft rejection in a combination of additional post-transplant immune suppression with tacrolimus and MMF. The optimal scenario in this setting would be to spare non-alloreactive donor naïve and memory T cells, both to guarantee primary responses to newly encountered antigens and, simultaneously, to confer adaptive immunity to the recipient.^90^ Using a non-myeloablative conditioning regimen of CY, fludarabine, and 2 Gy TBI and GVHD prophylaxis with PTCY (50 mg/kg days 3 and 4), MMF (days 5–35), and tacrolimus (days 5–180), a sustained engraftment was achieved in 87% of 210 acute leukemia patients treated by the Johns Hopkins group.^11^ Grade II–IV and III–IV aGVHD occurred in 27% and 5% of patients, respectively; cGVHD in 15%. The cumulative incidences of relapse and NRM were 55% and 18%, respectively. A total of 113 patients died of relapse (n=79), infections (n=15), pulmonary complications (n=7), GVHD (n=5), or other causes (n=7). Overall survival and event-free survival (EFS) at 3 years were 41% and 32%, respectively. The high relapse rate, which was probably the result of poor disease debulking by the nonmyeloablative conditioning and lack of GVHD-related GVL effect, dampened the advantage of a relatively low NRM. Switching to myeloablative conditioning regimens (MAC) reduces the risk of relapse but increases the NRM.^91^ In an attempt to reduce the risk of relapse, they adopted, in Genoa, a MAC regimen that consisted of either thiotepa, busulfan, fludarabine (TBF) or TBI and fludarabine (F-TBI).^37^ The choice of the conditioning was based on the patient’s age, restricting TBI to patients under the age of 56, and on whether they had already received a previous allogeneic graft with TBI in the conditioning. Three patients died before engraftment could be evaluated (one from Legionella pneumonia); the cumulative incidence of engraftment was 90% for neutrophils on day +32 and 86% for platelets on day +60. The CI rate of grade II–III aGVHD was 12%, and no patient developed grade IV aGVHD, and there was a 26% incidence of chronic GvHD. The cumulative incidence of TRM at 6 months was 18%, most of the events occurring early (median day +68), with 1 outlier on day +168. The CI rate of TRM was 9% for patients in remission and 26% for patients in relapse (P=0.10). Transplantation-related complications and infections were: hepatic sinusoidal obstructive syndrome in 1 patient (2%), severe mucositis in 3 patients (6%), hemorrhagic cystitis in 20 (40%), pericarditis in 4 (9%). BK virus was detected in 13 patients (65%). CMV reactivation occurred in 25 patients (50%) and CMV disease in 2 (1 colitis and 1 pneumonia) with a median time of CMV reactivation of 39 days (range, 3–60). EBV DNAemia was detected in 4 patients, HHV6 viremia in 1, adenovirus pneumonia in 1. Nineteen patients developed sepsis, occasionally with more than one causative agent: 9 gram-negative sepsis (6 E. coli, 1 Enterobacteriacae, 1 Corynebacterium, and 1 Pseudomonas aeruginosa); 13 gram-positive sepsis, most Streptococcus viridans and Enterococcus faecium. IFI were seen in 9 patients (5 Aspergillus pneumonia, 2 Candida krusei sepsis, and 1 Fusarium). Infections were the primary cause of death in 6 patients (12%), 3 sepsis and 3 pneumonia (Legionella, adenovirus, and Invasive aspergillosis). With a median follow-up of over 8 months (range, 4–22 months), the actuarial overall survival and DFS at 18 months from transplantation was 62% and 51%, respectively.

Crocchiolo et al.^38^ described infectious complications after unmanipulated haploHSCT using PTCY in 70 consecutive adult patients with lymphoma and found, aside from a high incidence of viral infections/reactivations, especially in the early post-transplant period, a quite low incidence of late bacterial infections, together with a very low incidence of IFIs after day +180. At last follow-up, a total of 224 documented infectious events occurred among 67 of 70 patients, with a median of 3 events/patient (range 1–10); 55% were of viral origin (n = 123), 40% bacterial (n = 89), 5% fungal (n = 11). Cumulative incidence of first viral infection was 70% and 77% at day +100 and +365, respectively; at one year, the incidence of bacterial infections and IFI were 63% and 12%, respectively. In 54% (35 of 65 patients at risk) at least one CMV reactivation developed; two non-fatal (1 colitis, 1 pneumonia) and 1 fatal (pneumonia) CMV diseases occurred. No primary CMV infections occurred in the 5 CMV seronegative patient/donor pairs. Polyomavirus-related hemorrhagic cystitis was observed in 13 patients (19%): 10 were caused by BK virus and 3 by JC virus. No EBV-related lymphoproliferative disorders occurred. Forty-five patients (64%) presented with at least one documented bacterial infection: 10 (14%), 21 (30%), and 14 (20%) patients had an infection by gram-positive, gram-negative, or both types of bacteria, respectively. Eleven IFIs were detected in 9 patients: n = 6 probable invasive aspergilloses (pneumonia in 5 patients and sinusitis in 1), n = 5 invasive candidiasis, all by non-albicans Candida (2 candidemias, 2 colitis, and 1 hepatosplenic candidiasis); median of occurrence was 62 days from haplo-HSCT (range 0–739). Notably, no cases occurred under active GVHD, and only 2 IFI occurred beyond day +180. When considering the timing of all episodes, bacterial infections occurred mostly between day 0 and +30, whereas viral infections/reactivations between days +31 and +100, with 11.08 bacterial events/1000 pt-days between day 0 and +30, and 15.15 viral events/1000 pt-days between days +31 and +100. The overall incidence of viral events between day 0 and day +180 was 8.8 events/1000 pt days. A total of 13 bacterial and 13 viral infections were observed after 1 year from transplant. These results are in line with that reported usually in the setting of unmanipulated haplo-HSCT. Ciurea et al.^92^ analyzed the early results of haplo-HSCT in adult recipients treated on 2 successive studies, and report improved outcomes with T cell replete (TCR) grafts and PTCY. Viruses were the most common cause of infection in both groups. During the first 180 days posttransplantation, there were 22 episodes per 1000 patient-days in the TCD group and 11 episodes per 1000 patient-days in the TCR group. Patients in the TCD group were 1.5 times more likely to develop a viral infection (P = 0.035) during this time period. Among the viral infections, CMV reactivation and human polyomavirus BK cystitis were the most frequent. Fourteen of 33 patients (42.2%) had CMV reactivation with a total of 30 episodes in the TCD group compared with 15 of 32 patients (46.8%) with 24 episodes in TCR group. For human polyomavirus BK cystitis, 15 of 33 cases (45.4%) were found in the TCD group versus 11 of 32 (34.4%) in the TCR group. There was no significant difference in the incidence of bacterial infections between the 2 groups. Thirty-two bacterial infections occurred in the TCR group compared with 38 episodes in the TCD group. On average, 7 and 10 episodes occurred per 1000 patient-days for the TCR and TCD group, respectively. Invasive fungal infections were third in frequency, with 12 episodes in 11 of 33 patients (33%) in the TCD group, and only 3 episodes in 3 of 32 patients (9%) in the TCR group. Patients in the TCD group were 5.6 times more likely to have an IFI within 6 months post-transplantation than those in the TCR group. Survival analysis revealed a significantly lower probability of death from an infection in the TCR group. The NRM attributed to infections was 24% in the TCD group and 9% in the TCR group (P = 0.01).

Similarly, Tischer et al.^93^ retrospectively compared the incidence of virus infections and outcome in the context of immune reconstitution in two different haplo-HSCT settings. The first was a combined T-cell-replete and T-cell-depleted approach using ATG over the conditioning (cTCR/TCD group, 28 patients; median age 31 years). The second was a T-cell-replete (TCR) approach using PTCY (TCR/PTCY group, 27 patients; median age 43 years). The incidence of herpesvirus infection was markedly lower in the TCR/PTCY (22%) than in the cTCR/TCD group (93%). Recovery of CD4+ T cells on day +100 was faster in the TCR/PTCY group. CMV reactivation was 30% in the TCR/PTCY compared to 57% in the cTCR/TCD group, and control with antiviral treatment was superior after TCR/PTCY transplantation (100 vs. 50 % cTCR/TCD). Furthermore, EBV-related lymphomas were observed only in the cTCR/TCD group (25% vs. 0%). Although the incidence of aGvHD grades II–IV and 1-yr overall survival were comparable in both groups, virus infection-related mortality was significantly lower after TCR/PTCY transplantation (0% vs 29%; p = 0.009). Cumulatively, 139 occurrences of virus infection were observed, 71 of them asymptomatic, 68 symptomatic, and 20 associated with disease. Virus infections affected 46 of 55 patients, whereas no virus infection was detected in nine patients only. Cumulatively, 87 occurrences of viral pathogens were identified in 27 patients (96 %) of the cTCR/TCD group. Within these occurrences, 51 patients were symptomatic or developed disease (n = 17). In the TCR/PTCY group, 52 occurrences were seen in 19 patients (70 %), 17 symptomatic or suffering from disease (n= 3). The most frequently observed viral pathogens across both groups were HHV-6, polyomavirus JC/BK, EBV, CMV, HSV, and ADV. In particular, symptomatic virus infection and disease were induced by the Herpesviridae HSV, VZV, CMV, EBV, and HHV-6 in 26 patients (93 %) of the cTCR/TCD group, but only in six patients (22 %) of the TCR/PTCY group. Besides the occurrence of HHV-6, for each of these Herpesviridae, the incidence of virus infection-related symptoms and disease was distinctly higher in the cTCD/TCR than in the TCR/PTCY group. On day + 100, predictors of better OS were lymphocytes >300/μl, CD3+ T cells >200/μl, and CD4+ T cells >150/μl, whereas the application of steroids >1 mg/kg was correlated with worse outcome.

These results suggest the strategy based on the infusion of unmanipulated graft followed by PTCY by preserving antiviral immunity and allowing fast immune recovery of CD4+ T cells is well suited to handle the important issue of infection after haplo-HSCT.

## Conclusions

Much progress has been made over the past 20 years in the clinical, biological and technical aspects of the T cell-depleted as well as of unmanipulated full-haplotype-mismatched HSCT. Haplo-HSCT has evolved from a last-attempt option for end-stage patients, to an established form of treatment that must be considered for selected patients with high-risk hematological disorders. Today, a high rate of engraftment can be achieved without severe GvHD and with low regimen-related toxicity and mortality. Although haplo-HSCT strategies differ according to each center and clinician, the current options include *in vitro* selective T cell-depleted “megadose” stem cell graft with no pharmacologic prophylaxis of GVHD or *in vivo* T cell depletion using the GIAC strategy or PTCY strategy (Table 3 ).

Improving immunologic reconstitution remains a major issue, as it represents the key to further decreasing toxicity and NRM in any form of transplantation. In the last decade, numerous advances in graft engineering and pharmacologic management of alloreactivity have decreased the incidences of GVHD and improved immune reconstitution offering the unique opportunity to harness both natural and adaptive immunity to control infections making this graft source an acceptable option for patients without a suitable matched donor.

## Figures and Tables

**Table 1:** HSCT outcomes in patients with acute myelogenous leukemia (haplo versus matched sibling donor-MSD, matched unrelated donor-MUD, and unrelated cord blood-UCB).

	HAPLO	MSD	MUD	UCB

Transplant-Related Mortality	18%	24%	33%	35%

CD4+ T cells/*μ*L at day +100	190	229	106	63

Cumulative incidence of CMV antigenaemia	74%	58%	60%	68%

Infection incidence at day +100:				
Bacterial	25%	23%	36%	39%
Fungal	11%	4%	14%	14%

Rate of fatal infections	11%	4%	14%	17%

**Table 2:** Invasive Fungal Infection in unmanipulated Haplo-HSCT.

# Pts	GvHD prophylaxis	IFI prophylaxis	IFI (%)	Reference
291	ATG, CSA, MTX, MMF	fluconazole	8.25 early phase 13.4 late phase	Sun et al. [35]
80	ATG, MTX, CSA, MMF, basiliximab	fluconazole	12.5 early phase 7 late phase	Di Bartolomeo et al [36]
50	PTCY, CSA, MMF	fluconazole	16	Raiola et al [37]
70	PTCY, MMF	micafungin	12	Crocchiolo [38]
26	PTCY, CSA/FK, MMF (RIC in Hodgkin disease)	fluconazole, or caspofungin followed by itraconazole	8	Raiola et al [39]

ATG: anti-thymocyte globulin. CSA: cyclosporine. MTX: methotrexate. MMF: mycophenolate mofetil. PTCY: post-transplant cyclophosphamide. FK: tacrolimus. RIC: reduced intensity conditioning. GVHD: graft vs host disease.

**Table 3:** Strategies to decrease infections after haplo-HSCT.

Revised T cell depleted HSCT	
Add back mature donor T cells with a broad repertoire	- Pathogen-specific T lymphocytes - Suicide gene insertion (HSV-TK) into T cells - iC9-modified T cells - Photodynamic purging of alloreactive T cells - Coinfusion of donor T-regs with conventional T cells
Selective T cell depletion	- CD3-CD19-depletion - αβ TCR/CD19-depletion - CD45RA-depletion
**Unmanipulated HSCT**	
Control of GVHD	- post-transplant Cyclophosphamide
Minimal extra-hematological toxicity	- Reduced intensity conditioning regimens
